# Anticoagulation Resumption in a Patient With Mechanical Heart Valves, Antithrombin Deficiency, and Hemorrhagic Transformation Following Thrombectomy After Ischemic Stroke

**DOI:** 10.3389/fphar.2020.549253

**Published:** 2020-12-16

**Authors:** Yi-Chen Li, Rong Wang, Hang Xu, Lan-Ping Ding, Wei-Hong Ge

**Affiliations:** ^1^ Department of Pharmacy, Nanjing University Medical School Affiliated Nanjing Drum Tower Hospital, Nanjing, China; ^2^ Department of Neurosurgery, Nanjing University Medical School Affiliated Nanjing Drum Tower Hospital, Nanjing, China; ^3^ Department of Cardio-Thoracic Surgery, Nanjing University Medical School Affiliated Nanjing Drum Tower Hospital, Nanjing, China; ^4^ Department of Pharmacy, First Affiliated Hospital of Nanjing Medical University, Nanjing, China

**Keywords:** anticoagulation, argatroban, hemorrhagic transformation (HT), valve thrombosis, thrombectomy

## Abstract

Anticoagulation is essential for patients undergoing mechanical heart valve replacement; however, the timing to reinitiate the anticoagulant could be a dilemma that imposes increased risk for bleeding events in patients suffering from the life-threatening hemorrhagic transformation (HT) after ischemic stroke. Such a situation was presented in this case report. A 71-year-old woman was transferred directly to the Neurocritical Care Unit because of a HT that occurred following the mechanical thrombectomy for ischemic stroke. Since she had a history of prosthetic metallic valve replacement, how the anticoagulating therapy could balance the hemorrhagic and thrombotic risks was carefully evaluated. On day 6 after the onset of hemorrhage transformation, the laboratory results of coagulation and fibrinolysis strongly suggested thrombosis as well as antithrombin deficiency. The short-acting and titratable anticoagulant argatroban was immediately initiated at low dose, and thrombosis was temporarily terminated. On day 3 of anticoagulation resumption, argatroban was discontinued for one dose when the prothrombin time and activated partial thromboplastin time significantly prolonged after argatroban infusion. Aortic valve thrombosis was detected the next day. The anticoagulation was then strengthened by dose adjustment to keep mitral valve intact, to stabilize the aortic valve thrombosis, and to decrease the aortic flow rate. The intravenous argatroban was transited to oral warfarin before the patient was discharged. This study is the first report of administering argatroban and titrating to its appropriate dose in the patient with valve thrombosis, antithrombin deficiency, and HT after mechanical thrombectomy for acute ischemic stroke. Notably, the fluctuations argatroban brings to the coagulation test results might not be interpreted as increased bleeding risk. This case also suggested that the reported timing (day 6 to day 14 after hemorrhage) of anticoagulant resumption in primary intracerebral hemorrhage with mechanical valves might be late for some patients with HT.

## Introduction

Patients with mechanical heart valves (MHV) require lifelong and sufficient anticoagulation; however, it becomes more complex when hemorrhagic events occur. Hemorrhagic transformation (HT) following ischemic stroke and reperfusion therapy, as one type of these bleeding events, occurs in a significant proportion of patients (Sussman and Connolly, 2013). The presented case scenario poses a therapeutic dilemma on the appropriate use of anticoagulants to maintain mechanical valve free of thrombosis meanwhile stabilizing HT hematoma volume to control bleeding event.

## Case Presentation

A 71-year-old Chinese woman was transferred to our Neurocritical Care Unit due to a progressive deterioration of consciousness for three consecutive days. Her medical history indicated a prosthetic aortic and mitral MHV replacement (tilting-disc valves) 24 years ago to restore her cardiac function impaired by rheumatic heart disease. To prevent valve thrombosis, warfarin was routinely administered after the surgery. Her international normalized ratio (INR) was periodically checked and maintained at the range of 1.8–2.5, which was optimal for Chinese patients (You et al., 2005; Zhang et al., 2012; Zhang et al., 2020). Three years ago, she developed atrial fibrillation and was implanted with a pacemaker. She was diagnosed with dermatomyositis last year and was treated with methylprednisolone and hydroxychloroquine. Six days prior to the admission, she initially suffered from back pain and was then admitted to the local hospital. Carotid ultrasound showed right carotid and right vertebral artery stenosis. The echocardiography showed moderate tricuspid regurgitation and atrial enlargement. The ejection fraction (EF) was 75%. Three days after her hospitalization, she complained about her impaired motor function of the left upper extremity and deterioration of the verbal ability and mental state. Computed tomography (CT) scan showed a dotted low density in the region of her basal ganglia and radiate corona. Subsequent digital subtraction angiography (DSA) discovered an occlusion in the M2 segment of the right middle cerebral artery, and the Alberta Stroke Program Early CT Score (ASPECTS) value was 7. An endovascular thrombectomy was performed. The modified treatment in cerebral ischemia (mTICI) score was graded 2a. The CT scan after the thrombectomy showed low density in right temporoparietal lobe, whereas the CT scan 24 h after the thrombectomy showed HT in the right temporal lobe. The HT was classified as parenchymal hematoma type 2 (PH2) (Sussman and Connolly, 2013), which is associated with worse clinical outcome (Zhang et al., 2014). The patient was intubated in the local hospital for hypoxemia. The National Institute of Health Stroke Scale (NIHSS) score was 28 before intubation. Since the onset of the hemorrhagic event, warfarin was discontinued and no INR reversal was utilized.

**FIGURE 1:**
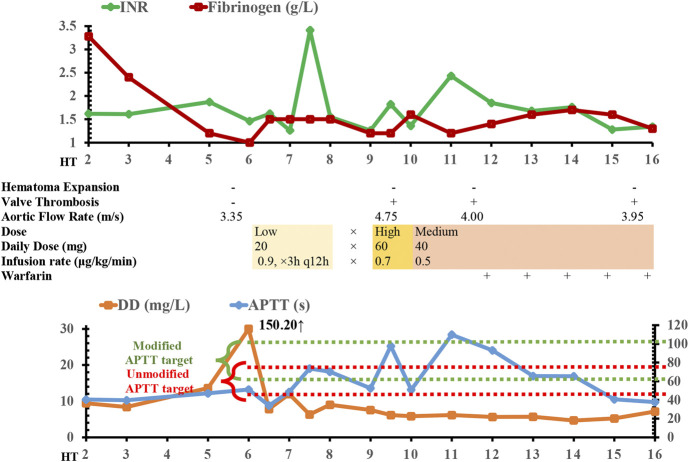
Time course of argatroban treatment, radiology, coagulation/fibrinolysis tests, and echocardiography findings. APTT, activated partial thromboplastin time; DD, D-dimer; INR, international normalized ratio; HT, hemorrhagic transformation; ×, discontinuation of argatroban.

**FIGURE 2:**
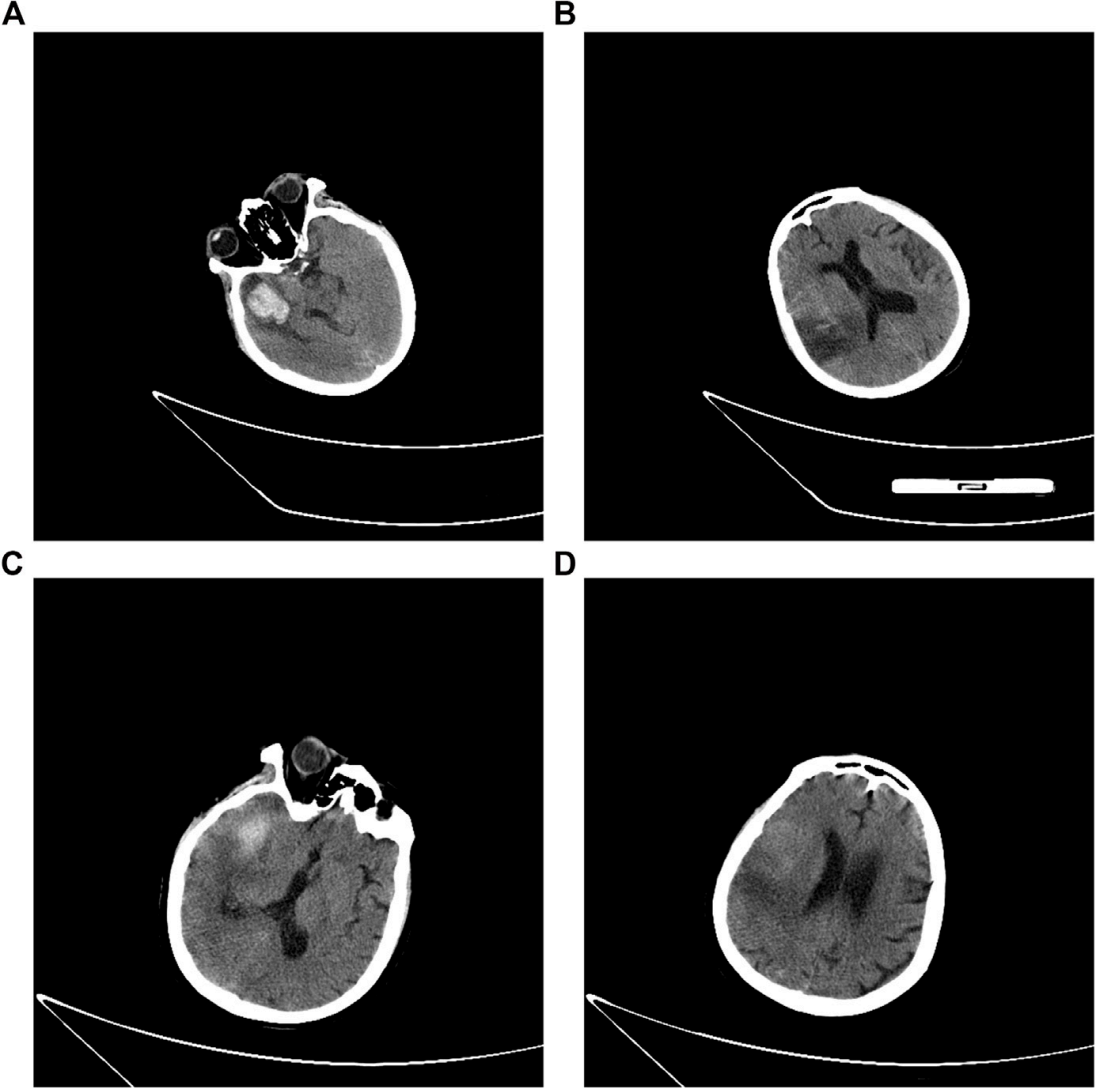
Head computed tomography scan on admission **(A, B)** and before discharge **(C, D)**.

The patient was transferred from the local hospital to our unit one day after her HT occurrence. On admission, the patient was mechanically ventilated (pO_2_ at 69 mmHg with F_i_O_2_ at 40%, without the assistance of ventilator), with a Glasgow Coma Scale score of 4T (E2VTM2). Blood tests on the day of her admission revealed the presence of hypoalbuminemia (serum albumin 31.9 g/L, normal range: 44–55 g/L) and a slightly elevated level of B-type natriuretic peptide (163 pg/ml, normal range: 5–100 pg/ml). Her platelet counts (90 × 10^9^/L, normal range: 125 × 10^9^/L to 350 × 10^9^/L), hemoglobin level (96 g/L, normal range: 130–175 g/L), and hematocrit (29.1%, normal range: 40–50%) were decreased. The level of antithrombin III (AT III, 46.0%, normal range: 103.2–113.8%) was reduced, and the levels of D-dimer (8.37 μg/ml, normal range: <0.5 μg/ml) and fibrin(-ogen) degradation product (FDP, 19.5 mg/L, normal range: 0–5 mg/L) were elevated. The prothrombin time (PT, normal range: 10–15 s) was 18.7 s and the level of INR was at 1.61. In the meantime, the activated partial thromboplastin time (APTT, normal range: 25–31.3 s), thrombin time (TT, normal range: 13–21 s), and her thromboelastography (TEG) indicated no abnormality (Figure 1). Her level of C-reactive protein was 60.8 mg/L (normal range: 0–8 mg/L). Her lab results showed normal liver function and renal function and normal level of electrolytes. The electrocardiography demonstrated the presence of atrial fibrillation, tachycardia, and ST–T-wave changes. Cerebral CT scan (Figures 2A,B) showed: 1) multiple acute infarcts in the right frontal, temporal parietal lobe, 2) temporal lobe hematoma, 3) right internal capsule infarct, 4) right cerebellar infarct, and 5) brain stem infarct. Echocardiography revealed: 1) moderate-to-severe tricuspid regurgitation, 2) right ventricular volume overload, and 3) mild-to-moderate pulmonary arterial hypertension. Aortic flow rate was 3.35 m/s, and no valve thrombosis was discovered at that time.

On day 6 of the onset of her hemorrhage, the level of her D-dimer was increased to 150.20 mg/L, and the level of fibrinogen (normal range: 2–4 g/L) was plummeted to 1.0 g/L. Her AT III level was as low as 40.3%. These results suggested a high risk of covert thrombotic event, although there was no overt symptom. After a detailed multidisciplinary discussion, we decided to reinitiate anticoagulation in a manageable way. The anticoagulant should possess relatively titratable pharmacokinetic-pharmacodynamic (PK-PD) relationship and could be discontinued when hemorrhagic event occurred. The drug heparin was excluded from the regime, as heparin targets ATIII (which was low) and thus was not the preferred option. Finally, the short-acting direct thrombin inhibitor argatroban was administered at the dose of 0.9 μg/kg/min lasting for 3 h twice a day (20 mg/d) to prevent fatal conditions especially valve thrombosis from happening. We utilized the maintenance dose of argatroban as the treatment was experimental, and the loading dose of 60 mg/d for 24 h continuous infusion was not chosen to avoid potential hematoma expansion. After one dose of argatroban, the level of fibrinogen was normalized to 1.5 g/L and D-dimer to 7.83 mg/L (Figure 1), which suggested the efficacy of anticoagulation resumption.

A coagulation function test was collected upon the end of argatroban infusion on day 7 of HT onset, which showed the PT at 40.2 s and INR at 3.41. The significantly elevated PT/INR implied a possibility that the hemorrhagic risk might outweigh the risk of thrombosis. Thus, argatroban was discontinued for 1 day. Unfortunately, the day after argatroban discontinuation, the echocardiograph demonstrated an aortic valve thrombosis leading to valvular insufficiency and a surge in aortic flow rate to 4.75 m/s (Figure 1). The mechanical mitral valve functioned normally. The echocardiograph also showed moderate-to-severe tricuspid regurgitation, right ventricular volume overload, and moderate pulmonary arterial hypertension. The patient was unstable to tolerate a transesophageal echocardiography for further verification of the thrombosis. The dose of argatroban was increased to 60 mg by continuous infusion for the first 24 h (0.7 μg/kg/min) and the infusion was maintained at 40 mg per day thereafter (0.5 μg/kg/min). At the same time, 4 units of cryoprecipitate (containing 300 mg of fibrinogen) were transfused when the level of fibrinogen dropped to 1.2 g/L the second time.

On day 13, her echocardiography showed that the aortic flow rate was decreased to 4.0 m/s, although the aortic valve thrombosis still presented. Considering the absence of hematoma expansion within the brain, verified by head CT, warfarin was initiated to bridge the intravenous argatroban. The overlap continued for 5 days. The patient was then discharged and transferred to the local rehabilitation hospital with the residual symptoms of consciousness impairment (Glasgow Coma Scale, 9T: E4VTM5, tracheotomized) and mild left-sided hemiparesis (muscle strength of left extremities: level 1; right: level 3). The CT scan before her discharge (Figures 2C,D) showed a reduction of hematoma volume; the CT perfusion demonstrated that the regional cerebral blood volume (CBV) and cerebral blood flow (CBF) were reduced in the right temporoparietal lobe while mean transit time (MTT) and time to peak (TPP) prolonged.

Following up the patient 3 months after discharge, her modified Rankin Scale (mRS) was four and Glasgow Outcome Scale (GOS) was three. The echocardiography revealed no valve thrombosis and the EF was 65%.

## Discussion

The risk of HT deterioration with an indication for anticoagulation after acute ischemic stroke is multifactorial (Marsh et al., 2013; László and Hortobágyi, 2017). Endovascular therapy for these stroke patients could also make them suffer from hemorrhage, particularly in patients with atrial fibrillation (Sussman and Connolly, 2013; Nogueira et al., 2015), and HT after thrombectomy was of comparatively high rate in patients taking anticoagulants (Kaesmacher et al., 2017; Purrucher et al., 2016). However, inadequate anticoagulation increases the risk of thrombotic obstruction of the tilting-disc valves, which could even be fatal (Ryder et al., 1984). Therefore, the timing and regimen of anticoagulation resumption after HT are more complex, as the individual’s condition, valve type, hematoma volume, lab results, and the pharmacology of the anticoagulants should all be taken into consideration. In the present case, we described the argatroban therapy initiation, dose adjustment, and transition to warfarin in a patient with MHV and HT following thrombectomy.

Previous study demonstrated that restarting therapeutic anticoagulation in anticoagulation-associated intracerebral hemorrhage (ICH) patients within 2 weeks is associated with increased hemorrhagic risk, and the earliest starting point of anticoagulant reinitiation is on day 6 for patients with high thromboembolic risk (Kuramatsu et al., 2018). However, HT presents with lower intracranial hemorrhage risk than ICH (da Silva and Frontera, 2017). In our case, the patient was anticoagulated on day 6, as thrombotic event was strongly suspected. However, the insufficient argatroban anticoagulation was also followed by aortic valve thrombosis. Therefore, unlike the ICH data, the optimal duration of nonanticoagulated period of HT patients with MHV might be shorter than that of the ICH patients.

Why did we administer argatroban instead of heparin to the patient? From the pharmacological perspective, argatroban (and bivalirudin) inhibits thrombin directly, alleviates thrombin-induced hypofibrinogenemia and thrombocytopenia dose-dependently, and rapidly prevents thrombosis. More importantly, due to the short half-life (<30 min) of argatroban, it would not be difficult to reverse the anticoagulant effect by immediate discontinuation of its infusion if a major bleeding occurred (Bush, 1991). The second reason we chose argatroban was based on the patient’s low level of antithrombin. Heparin interacts with antithrombin to exert its pharmacological effect (Loftus, 2016). Inadequate level of antithrombin (antithrombin deficiency, antithrombin level at 25–60% of the normal value) may result in diminished heparin efficacy, and heparin thus might not prevent or treat thrombosis at its regular dose (Fukuda et al., 2013). Antithrombin III is a critical glycoprotein, regulating coagulation by inhibiting thrombin (factor IIa) (Ornaghi et al., 2014). Acquired antithrombin deficiency, as an autoimmune disorder, is commonly due to increased coagulation activity secondary to endothelial injury or the presence of antiphospholipid antibodies (Mo and Bao, 2017). In these situations, the excessive activation of the coagulation system accelerates the consumption of antithrombin III (Büller and ten Cate, 1989). In the presented case, the patient, with 1-year history of dermatomyositis and discontinued medication since her recent stroke, presented thrombosis and decreased functional antithrombin level, ranging from 36.4 to 55.0%, indicating an acquired antithrombin deficiency. With continuous infusion of argatroban at a relatively low dose for the first 3 h, the D-dimer became gradually normalized. The action was rapid and efficient.

Notably, the argatroban increases PT/INR, APTT, and TT in a dose-dependent manner (Bush, 1991). APTT/INR elevation might be falsely interpreted as overanticoagulation, and the valve thrombosis might be the result of the misleading “PTT/INR confounding” (Warkentin, 2014). Traditionally, the target APTT range is 1.5–2.5 times normal APTT. However, the baseline APTT should also be taken into consideration (Warkentin, 2014). In the present case, baseline APTT of the patient (40.4 s) was higher than the normal range (25–31.3 s), and the APTT target should be simultaneously modified from (47.3–78.8 s) to (60.6–101 s). When we intermittently infused argatroban in the patient, we temporarily discontinued argatroban when the APTT increased to 73.7 s and INR to 3.41 at the end of argatroban infusion. Although the APTT at 73.7 s was at the upper limit of target and implying a higher bleeding risk, it was well within the modified target when baseline APTT was included to set the therapeutic range. The unmodified range falsely suggested that the patient is at risk of overanticoagulation, and the discontinuation of argatroban for one day might be associated with the subsequent valve thrombosis. In fact, the APTT/INR could not reflect the function of coagulation system because it would fluctuate along with the argatroban infusion. Hemodilution and the history of autoimmune disease could also contribute to the “PTT confounding” during argatroban treatment (Warkentin, 2014), and lupus anticoagulant should also be tested when anticoagulation reinitiation was considered. To conclude, the underestimation of the influence of argatroban on test results might cause an overestimation of the hemorrhagic risk and unnecessary discontinuation of argatroban. After the aortic valve thrombosis was verified, an increase in argatroban dose linearly strengthened the anticoagulant effect and ultimately improved the aortic flow rate.

The covert thrombosis of the patient had been progressing on day 6, indicating that her HT was not that “hemorrhagic” as anticoagulation-associated ICH, and her timing of anticoagulant resumption might be as early as day 6 of HT onset, or even earlier. The early initiation of argatroban infusion had significantly reversed the thrombotic event; however, the misleading PT/APTT results caused possibly unnecessary reduction of argatroban dosage and subsequent valve thrombosis.

The case report indicated a potential role of short-acting direct thrombin inhibitor in treating such patients in the acute phase, especially those with antithrombin deficiency. It also raised questions about the differentiated decision-making in the timing of anticoagulant resumption in HT and ICH patients, as HT and ICH, respectively, possess lower and higher intracranial hemorrhage risks (da Silva and Frontera, 2017). The “PTT/INR confounding” should be carefully evaluated when direct thrombin inhibitors are utilized, and underanticoagulation it brings might result in serious and fatal complications. Further study is warranted to shed light on the efficacy and safety of the thrombin inhibitors on HT patients with MHV, and disparity in thromboembolic risk of different valve types (e.g., tilting-disc and bileaflet valves) should also be taken into consideration.

## Data Availability Statement

The original contributions presented in the study are included in the article/Supplementary Material; further inquiries can be directed to the corresponding author.

### Ethics Statement

Written informed consent was obtained from the individual's legal guardian for the publication of any potentially identifiable images or data included in this article.

## Author Contributions

YL, RW, HX, and LD participated in the critical care of the patient. YL, RW, and WG wrote the manuscript. HX and RW revised the manuscript.

## Conflict of Interest

The authors declare that the research was conducted in the absence of any commercial or financial relationships that could be construed as a potential conflict of interest.
